# A new polymorph of magnesium oxalate dihydrate

**DOI:** 10.1107/S1600536808015870

**Published:** 2008-06-07

**Authors:** Xue-An Chen, Fang-Ping Song, Xin-An Chang, He-Gui Zang, Wei-Qiang Xiao

**Affiliations:** a College of Materials Science and Engineering, Beijing University of Technology, Ping Le Yuan 100, Beijing 100124, People’s Republic of China; b Institute of Microstructure and Properties of Advanced Materials, Beijing University of Technology, Ping Le Yuan 100, Beijing 100124, People’s Republic of China

## Abstract

In the asymmetric unit of the title compound, *catena*-poly[[diaqua­magnesium(II)]-μ-oxalato], [Mg(C_2_O_4_)(H_2_O)_2_]_*n*_, there is one Mg atom in an octa­hedral coordination with site symmetry 222, a unique C atom of the oxalate anion lying on a twofold axis, an O atom of the anion in a general position and a water O atom at a site with imposed twofold rotation symmetry. The Mg^2+^ ions are ligated by water mol­ecules and bridged by the anions to form chains that are held together by O—H⋯O hydrogen bonds. The structure of the title compound has already been reported in a different space group [Lagier, Pezerat & Dubernat (1969 ▶). *Rev. Chim. Miner.* **6**, 1081–1093; Levy, Perrotey & Visser (1971 ▶). *Bull. Soc. Chim. Fr.* pp. 757–761].

## Related literature

For related literature, see: Basso *et al.* (1997 ▶); Caric (1959 ▶); Deyrieux *et al.* (1973 ▶); Echigo *et al.* (2005 ▶); Huang & Mak (1990 ▶); Lagier *et al.* (1969 ▶); Le Page (1987 ▶); Lethbridge *et al.* (2003 ▶); Levy *et al.* (1971 ▶); Neder *et al.* (1997 ▶); Schefer & Grube (1995 ▶); Tazzoli & Domeneghetti (1980 ▶); Vanhoyland, Bouree *et al.* (2001 ▶); Vanhoyland, Van Bael *et al.* (2001 ▶). 
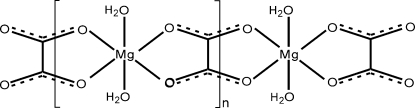


## Experimental

### 

#### Crystal data

[Mg(C_2_O_4_)(H_2_O)_2_]
*M* *_r_* = 148.36
Orthorhombic, 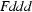

*a* = 5.3940 (11) Å
*b* = 12.691 (3) Å
*c* = 15.399 (3) Å
*V* = 1054.1 (4) Å^3^
*Z* = 8
Mo *K*α radiation
μ = 0.29 mm^−1^
*T* = 290 K
0.30 × 0.20 × 0.15 mm

#### Data collection

Rigaku AFC-7R diffractometer
Absorption correction: ψ scan (Kopfmann & Huber, 1968 ▶) *T*_min_ = 0.915, *T*_max_ = 0.962
1110 measured reflections
483 independent reflections
321 reflections with *I* > 2σ(*I*)
*R*_int_ = 0.054
3 standard reflections every 150 reflections intensity decay: 1.1%

#### Refinement

*R*[*F* ^2^ > 2σ(*F* ^2^)] = 0.034
*wR*(*F* ^2^) = 0.110
*S* = 0.97
483 reflections
27 parameters
All H-atom parameters refined
Δρ_max_ = 0.89 e Å^−3^
Δρ_min_ = −0.48 e Å^−3^

### 

Data collection: *Rigaku/AFC Diffractometer Control Software* (Rigaku, 1994 ▶); cell refinement: *Rigaku/AFC Diffractometer Control Software*; data reduction: *Rigaku/AFC Diffractometer Control Software*; program(s) used to solve structure: *SHELXS97* (Sheldrick, 2008 ▶); program(s) used to refine structure: *SHELXL97* (Sheldrick, 2008 ▶); molecular graphics: *ATOMS* (Dowty, 1999 ▶); software used to prepare material for publication: *SHELXL97*.

## Supplementary Material

Crystal structure: contains datablocks global, I. DOI: 10.1107/S1600536808015870/pv2083sup1.cif

Structure factors: contains datablocks I. DOI: 10.1107/S1600536808015870/pv2083Isup2.hkl

Additional supplementary materials:  crystallographic information; 3D view; checkCIF report

## Figures and Tables

**Table 1:** Hydrogen-bond geometry (Å, °)

*D*—H⋯*A*	*D*—H	H⋯*A*	*D*⋯*A*	*D*—H⋯*A*
O2—H2⋯O1^i^	0.84 (3)	1.97 (2)	2.761 (1)	158 (2)

Symmetry code: (i) 
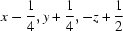
.

**Fig. 1.**
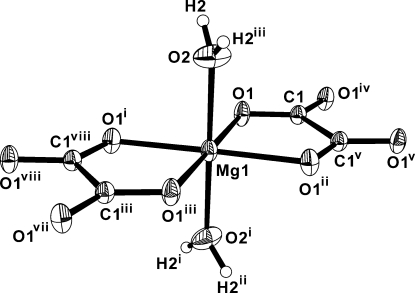
The coordination geometry of Mg1 in (I) with displacement ellipsoids drawn at the 50% probability level. Symmetry codes: (i) 3/4 - x, 3/4 - y, z; (ii) x, 3/4 - y, 3/4 - z; (iii) 3/4 - x, y, 3/4 - z; (iv) 7/4 - x, 3/4 - y, z; (v) 7/4 - x, y, 3/4 - z; (vii) -1 + x, 3/4 - y, 3/4 - z; (viii) -1 + x, y, z.

**Fig. 2.**
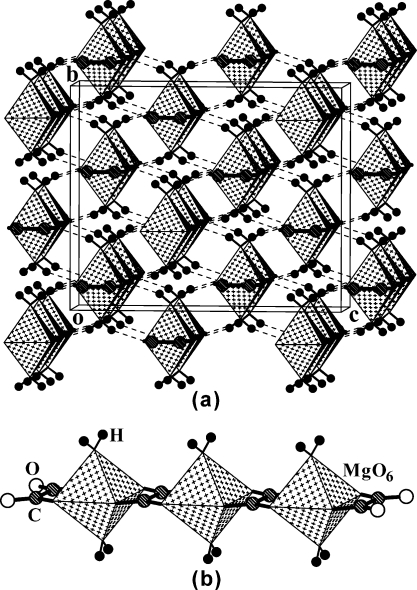
The crystal structure of (I) projected approximately along the [100] direction (a) as well as the single chain of [Mg(C2O4)(H2O)2] (b); the H2···O1 contacts are shown as dashed lines.

## supplementary crystallographic information

### Comment

Oxalates are of considerable interest because many of them are natural minerals and in addition, the oxalate anion can adopt different coordination modes to bind metals to form infinite chains, sheets and networks, leading to the rich structural chemistry. For instance, in the system of MO (M = alkali-earth metal)–H_2_C_2_O_4_–H_2_O, at least eight phases have been structurally characterized, including Mg(C_2_O_4_).2H_2_O (Lagier *et al.*, 1969; Levy *et al.*, 1971), Ca(C_2_O_4_).H_2_O (Echigo *et al.*, 2005), Ca(C_2_O_4_).2.375H_2_O (Tazzoli & Domeneghetti, 1980), Ca(C_2_O_4_).3H_2_O (Basso *et al.*, 1997), SrH(C_2_O_4_)(C_2_O_4_)_0.5_ (Vanhoyland, Van Bael *et al.*, 2001), SrH(C_2_O_4_)(C_2_O_4_)_0.5_.H_2_O (Vanhoyland, Bouree *et al.*, 2001), Ba(C_2_O_4_).H_2_O (Huang & Mak, 1990), and Ba(C_2_O_4_).3.5H_2_O (Neder *et al.*, 1997). Among them, Mg(C_2_O_4_).2H_2_O (which we call α-Mg(C_2_O_4_).2H_2_O later) was reported to have one-dimensional (1D) magnesium oxalate chains, Ca(C_2_O_4_).3H_2_O has a layered structure, and others contain three-dimensional metal oxalate frameworks. During our exploratory syntheses of novel hydrated borate materials, we have unexpectedly obtained a new Mg(C_2_O_4_).2H_2_O polymorph [called β-Mg(C_2_O_4_).2H_2_O in this work], (I), as a byproduct. It crystallizes in a space group different from those of other oxalates of similar stoichiometry. We describe its synthesis and crystal structure here for the first time.

The title structure contains Mg^2+^ cations, [C_2_O_4_]^2-^ anions, and H_2_O molecules as the fundamental structural building units (Fig. 1). The anions are bridged by octahedral Mg^2+^ centers to generate a 1D infinite polymeric chain, and H_2_O molecules are located on the two sides of the chains and coordinated to the Mg^2+^ centers to complete the octahedral coordination sphere (Fig. 2). The [Mg(C_2_O_4_)(H_2_O)_2_] chains extend along the [100] direction, and are held together *via* O—H···O hydrogen bonds (Table 1).

The Mg atom occupies one crystallographically distinct octahedral site with site symmetry 222. Each Mg^2+^ is coordinated by six O atoms, four of which are from two oxalate ions and the others from two H_2_O molecules. The Mg—O distances are very reasonable when compared with those observed in Mg(NO_3_)_2_.6H_2_O, where octahedrally coordinated Mg^2+^ is also found (Schefer & Grube, 1995). The unique C atom of the anion lies on a 2-fold axis and an O-atom on a general position. The unique O atom of H_2_O lies on a 2-fold axis. The oxalate ion is nearly planar, with a mean deviation of 0.0134 Å, and the bond geometries of [C_2_O_4_]^2-^ are in accord with those observed in other oxalate compounds (Lethbridge *et al.*, 2003).

In the previously reported oxalates, no one is exactly isotypic with the title compound. Several compounds including M(C_2_O_4_).2H_2_O (M = Mg, Fe, Co, Ni, Zn) (Levy *et al.*, 1971; Caric, 1959; Deyrieux *et al.*, 1973) also contain topologically identical [M(C_2_O_4_)(H_2_O)_2_] chains, but crystallize in the monoclinic space group *C2/c*. An examination of positional parameters of these compounds using the program MISSYM (Le Page, 1987) did not show potential additional symmetry. In fact, the space group *C2/c* of α-Mg(C_2_O_4_).2H_2_O as well as the isostructural analogs is a "*translationengleiche*" subgroup (index 2) of the group *Fddd* adopted by β-Mg(C_2_O_4_).2H_2_O. The lattice vectors of α-Mg(C_2_O_4_).2H_2_O (a_1_, a_2_ and a_3_) are related to those of its β-form (a, b and c) in the following manner: a_1_ = b, a_2_ = a, and a_3_ = -0.5b - 0.5c. The other compound, Mn(C_2_O_4_).2H_2_O, was also reported to exist in two forms. The α-phase (Deyrieux *et al.*, 1973) is isostructural with α-Mg(C_2_O_4_).2H_2_O, while the β-phase crystallizes in the space group *P2_1_2_1_2_1_* (Lethbridge *et al.*, 2003). The crystal structure of β-Mn(C_2_O_4_).2H_2_O also consists of chains of oxalate-bridged Mn^2+^ centers, but MnO_6_ octahedra in these chains are interconnected through sharing O corners and each oxalate ion acts as a tri-dentate ligand. This is different from the situation in other members of the M(C_2_O_4_).2H_2_O family of compounds, where MO_6_ octahedra are separated from each other and the oxalate ions act as tetra-dentate ligands. It is the difference in the coordination modes of the oxalate ions that is responsible for the structural versatility of M(C_2_O_4_).2H_2_O.

### Experimental

β-Mg(C_2_O_4_).2H_2_O was first obtained from a hydrothermal reaction in an attempt to prepare novel hydrated borates. For the preparation of MgB_6_O_10_, a stoichiometric mixture of MgO and B_2_O_3_ was heated at 873 K for two weeks with several intermediate re-mixings and the resulting product was identified to be the pure phase of MgB_6_O_10_ based on the powder XRD analysis. A 0.300 g (3.376 mmol) sample of MgB_6_O_10_, 3 ml pyridine, 0.5 ml 14.5 M (65%) HNO_3_, and 0.5 ml H_2_O were sealed in an 15-ml Teflon-lined autoclave and subsequently heated at 453 K for one week, then cooled slowly to room temperature. The product consisted of colorless, block-like crystals with the largest having dimensions of 0.6 × 0.6 × 0.8 mm^3^ in pale yellow mother liquor. The final pH of the reaction system was about 1.0. The crystals were isolated in about 30% yield (based on Mg) by washing the reaction product with deionized water and anhydrous ethanol followed by drying with anhydrous acetone. X-ray structural analysis indicated that the formula of this compound may be Mg(C_2_O_4_).2H_2_O. It is unclear how the oxalate groups are formed.

Subsequently, a separate set of experiments was conducted, in which the starting materials were: 0.2718 g (6.7403 mmol) MgO, 0.8497 g (6.7400 mmol) H_2_(C_2_O_4_).2H_2_O, and 3 ml H_2_O, and the heating and isolation procedures were the same as those described above. The reaction resulted in pure colorless crystals. The powder XRD pattern of the ground crystals in this experiment was in good agreement with that calculated from the single-crystal data of Mg(C_2_O_4_).2H_2_O from the former experiment, confirming that the same phase had been obtained.

### Refinement

H-atom positions were located in a difference Fourier map and all associated parameters were refined freely.

### Crystal data

**Table:**

[Mg(C_2_O_4_)(H_2_O)_2_]	*F*_000_ = 608
*M**_r_* = 148.36	*D*_x_ = 1.870 Mg m^−^^3^
Orthorhombic, *F**d**d**d*	Mo *K*α radiation λ = 0.71073 Å
Hall symbol: -F 2uv 2vw	Cell parameters from 25 reflections
*a* = 5.3940 (11) Å	θ = 13.0–19.6º
*b* = 12.691 (3) Å	µ = 0.29 mm^−^^1^
*c* = 15.399 (3) Å	*T* = 290 K
*V* = 1054.1 (4) Å^3^	Block, colourless
*Z* = 8	0.30 × 0.20 × 0.15 mm

### Data collection

**Table:**

Rigaku AFC-7R diffractometer	*R*_int_ = 0.054
Radiation source: fine-focus sealed tube	θ_max_ = 32.5º
Monochromator: graphite	θ_min_ = 4.2º
*T* = 290 K	*h* = 0→8
2θ/ω scans	*k* = 0→19
Absorption correction: ψ scan(Kopfmann & Huber, 1968)	*l* = 0→23
*T*_min_ = 0.915, *T*_max_ = 0.962	3 standard reflections
1110 measured reflections	every 150 reflections
483 independent reflections	intensity decay: 1.1%
321 reflections with *I* > 2σ(*I*)	

### Refinement

**Table:**

Refinement on *F*^2^	Secondary atom site location: difference Fourier map
Least-squares matrix: full	Hydrogen site location: difference Fourier map
*R*[*F*^2^ > 2σ(*F*^2^)] = 0.034	All H-atom parameters refined
*wR*(*F*^2^) = 0.110	*w* = 1/[σ^2^(*F*_o_^2^) + (0.0683*P*)^2^] where *P* = (*F*_o_^2^ + 2*F*_c_^2^)/3
*S* = 0.97	(Δ/σ)_max_ < 0.001
483 reflections	Δρ_max_ = 0.89 e Å^−^^3^
27 parameters	Δρ_min_ = −0.48 e Å^−^^3^
Primary atom site location: structure-invariant direct methods	Extinction correction: none

### Special details

**Table:**

Geometry. All esds (except the esd in the dihedral angle between two l.s. planes) are estimated using the full covariance matrix. The cell esds are taken into account individually in the estimation of esds in distances, angles and torsion angles; correlations between esds in cell parameters are only used when they are defined by crystal symmetry. An approximate (isotropic) treatment of cell esds is used for estimating esds involving l.s. planes.
Refinement. Refinement of F^2^ against ALL reflections. The weighted R-factor wR and goodness of fit S are based on F^2^, conventional R-factors R are based on F, with F set to zero for negative F^2^. The threshold expression of F^2^ > 2sigma(F^2^) is used only for calculating R-factors(gt) etc. and is not relevant to the choice of reflections for refinement. R-factors based on F^2^ are statistically about twice as large as those based on F, and R- factors based on ALL data will be even larger.

### Fractional atomic coordinates and isotropic or equivalent isotropic displacement parameters (Å^2^)

**Table:**

	*x*	*y*	*z*	*U*_iso_*/*U*_eq_	
Mg1	0.3750	0.3750	0.3750	0.0163 (2)	
C1	0.8750	0.3750	0.32406 (10)	0.0153 (3)	
O1	0.66783 (15)	0.37630 (11)	0.28779 (5)	0.0202 (3)	
O2	0.3750	0.53689 (11)	0.3750	0.0343 (4)	
H2	0.399 (6)	0.578 (2)	0.3335 (16)	0.050 (7)*	

### Atomic displacement parameters (Å^2^)

**Table:**

	*U*^11^	*U*^22^	*U*^33^	*U*^12^	*U*^13^	*U*^23^
Mg1	0.0117 (4)	0.0237 (4)	0.0136 (4)	0.000	0.000	0.000
C1	0.0149 (6)	0.0199 (6)	0.0110 (6)	−0.0001 (8)	0.000	0.000
O1	0.0145 (4)	0.0342 (5)	0.0120 (4)	0.0012 (4)	−0.0014 (3)	−0.0018 (4)
O2	0.0631 (11)	0.0228 (7)	0.0169 (6)	0.000	0.0086 (10)	0.000

### Geometric parameters (Å, °)

**Table:**

Mg1—O2^i^	2.0546 (15)	Mg1—O1	2.0734 (9)
Mg1—O2	2.0546 (15)	C1—O1	1.2494 (11)
Mg1—O1^i^	2.0734 (9)	C1—O1^iv^	1.2494 (11)
Mg1—O1^ii^	2.0734 (9)	C1—C1^v^	1.569 (3)
Mg1—O1^iii^	2.0734 (9)	O2—H2	0.84 (3)
			
O2^i^—Mg1—O2	180.0	O2^i^—Mg1—O1	90.45 (4)
O2^i^—Mg1—O1^i^	89.55 (4)	O2—Mg1—O1	89.55 (4)
O2—Mg1—O1^i^	90.45 (4)	O1^i^—Mg1—O1	99.26 (5)
O2^i^—Mg1—O1^ii^	89.55 (4)	O1^ii^—Mg1—O1	80.75 (5)
O2—Mg1—O1^ii^	90.45 (4)	O1^iii^—Mg1—O1	179.09 (8)
O1^i^—Mg1—O1^ii^	179.09 (7)	O1—C1—O1^iv^	126.89 (14)
O2^i^—Mg1—O1^iii^	90.45 (4)	O1—C1—C1^v^	116.56 (7)
O2—Mg1—O1^iii^	89.55 (4)	O1^iv^—C1—C1^v^	116.56 (7)
O1^i^—Mg1—O1^iii^	80.75 (5)	C1—O1—Mg1	113.06 (8)
O1^ii^—Mg1—O1^iii^	99.26 (5)	Mg1—O2—H2	128.7 (19)

Symmetry codes: (i) −*x*+3/4, −*y*+3/4, *z*; (ii) *x*, −*y*+3/4, −*z*+3/4; (iii) −*x*+3/4, *y*, −*z*+3/4; (iv) −*x*+7/4, −*y*+3/4, *z*; (v) −*x*+7/4, *y*, −*z*+3/4.

### Hydrogen-bond geometry (Å, °)

**Table:**

*D*—H···*A*	*D*—H	H···*A*	*D*···*A*	*D*—H···*A*
O2—H2···O1^vi^	0.84 (3)	1.97 (2)	2.761 (1)	158 (2)

Symmetry codes: (vi) *x*−1/4, *y*+1/4, −*z*+1/2.
